# Supplementary material for: An interaction network of inner centriole proteins organised by POC1A-POC1B heterodimer crosslinks ensures centriolar integrity
Source: Nat Commun. 2024 Nov 14;15:9857. doi: 10.1038/s41467-024-54247-5 (PMC11564547; doi:10.1038/s41467-024-54247-5)
Supplement: Supplementary file 1 — Supplementary Information [file 41467_2024_54247_MOESM1_ESM.pdf]

**a**

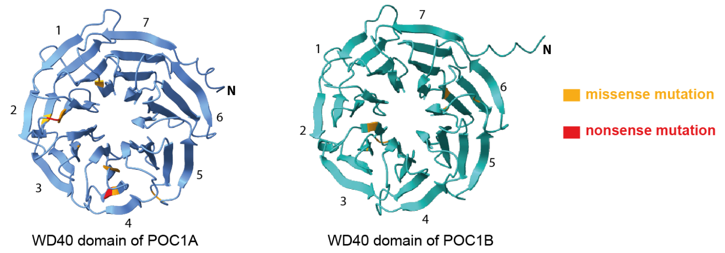


| Reported mutations of the WD40 domain in patients | |
|---|---|
| POC1A | POC1B |
| p.Val22Phe | p.Arg106Pro |
| p.Ser80Phe | p.Asp113His |
| p.Val85Leu | p.Thr119Ile |
| p.Thr120Ala | p.His227Arg |
| p.Ser164Asn | p.His237Arg |
| p.Leu171Pro | p.Thr246Met |
| p.Arg217Tyr | |
| p.Arg81* | |
| p.Try172* | |

**b**

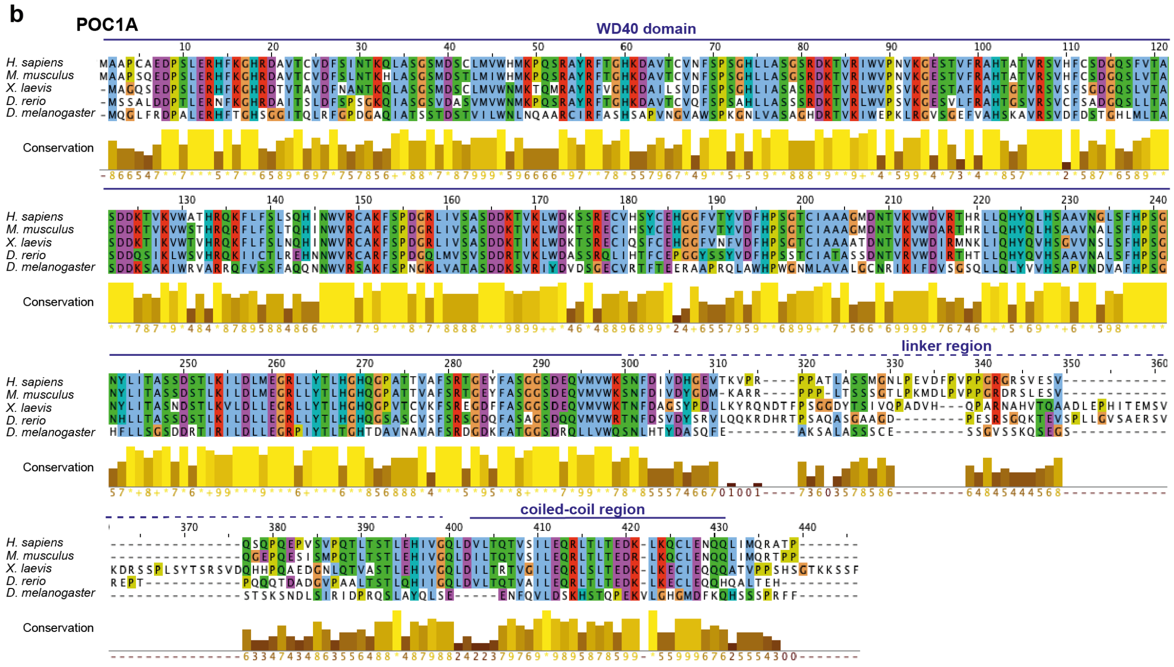


**c**

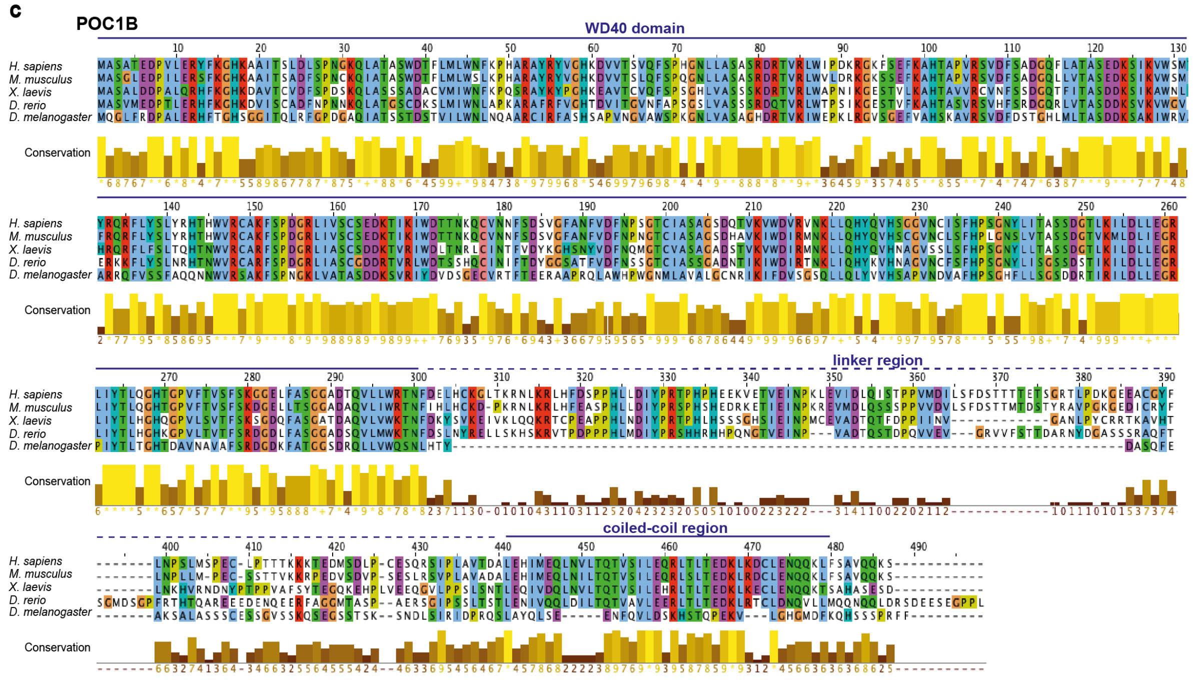


**Supplementary Figure 1. Patient mutations and conservation of POC1 proteins in different species**

**a** Disease-linked mutations in the WD40 domains of POC1A and POC1B found in patients[1-12]. Orange marks missense mutations, whereas red indicates nonsense mutations leading to a premature stop codon. **b, c** Alignment of POC1A and POC1B amino acid sequences across different species including the non-vertebrates POC1 from *Drosopholia melanogaster*. WD40 domains and coiled-coil regions show high conservation in these species. UniProtKB accession numbers of POC1A homologues: Q8NBT0 (*H. sapiens*), Q8JZX3 (*M. musculus*), Q7T0P4 (*X. laevis*), Q7ZVF0 (*D. rerio*). UniProtKB accession numbers of POC1B homologues: Q8TC44 (*H. sapiens*), Q8BHD1 (*M. musculus*), Q4V7Z1 (*X. laevis*), A2CEH0 (*D. rerio*). POC1: Q9VU65 (*D. melanogaster*).

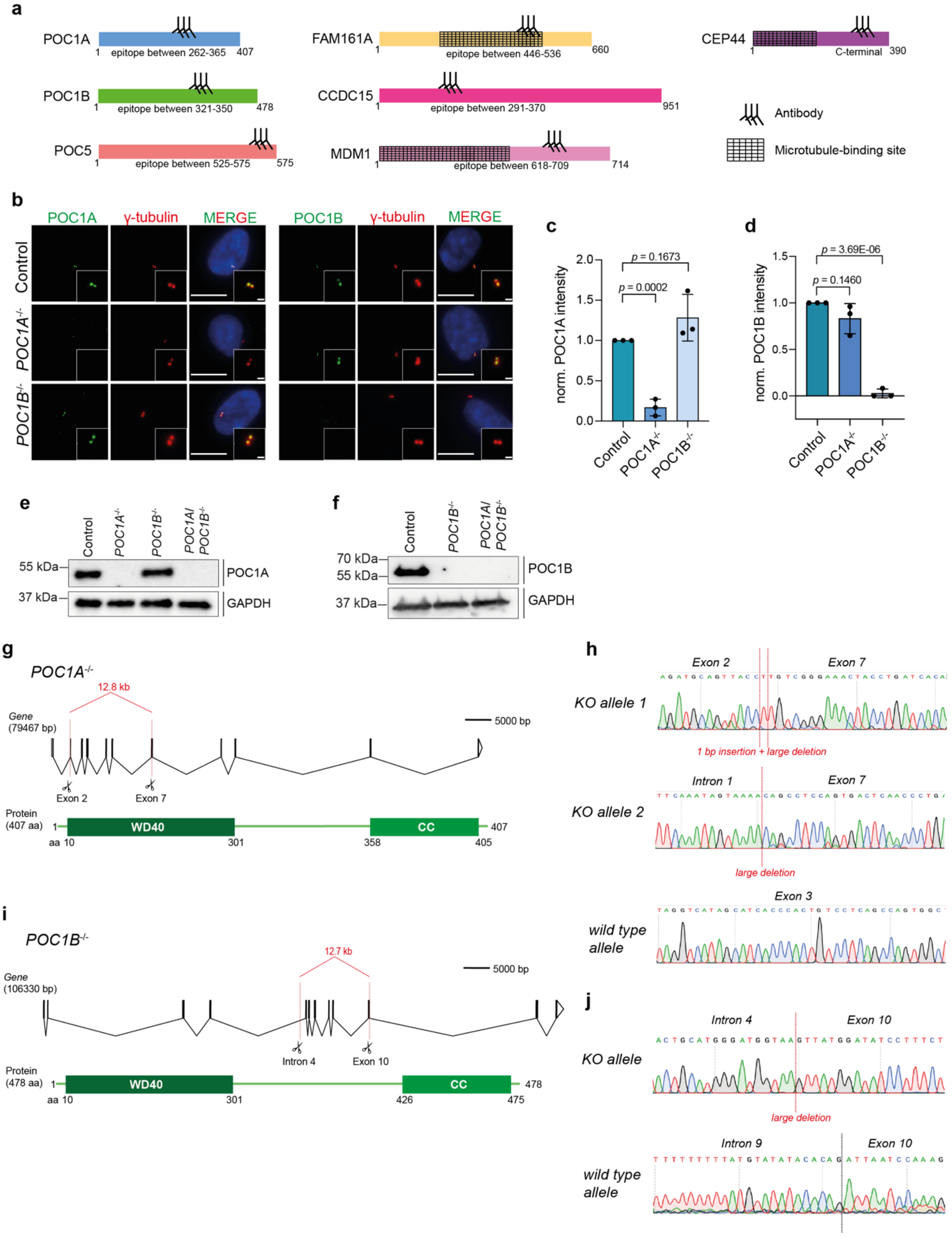
a
POC1A
epitope between 262-365
407
FAM161A
epitope between 446-536
660
CEP44
C-terminal
390
POC1B
epitope between 321-350
478
CCDC15
epitope between 291-370
951
POC5
epitope between 525-575
575
MDM1
epitope between 618-709
714
Antibody
Microtubule-binding site
b
POC1A
γ-tubulin
MERGE
POC1B
γ-tubulin
MERGE
Control
POC1A-/-
POC1B-/-
c
norm. POC1A intensity
p = 0.1673
p = 0.0002
Control
POC1A-/-
POC1B-/-
d
norm. POC1B intensity
p = 3.69E-06
p = 0.1460
Control
POC1A-/-
POC1B-/-
e
Control
POC1A-/-
POC1B-/-
POC1A/ POC1B-/-
55 kDa
37 kDa
POC1A
GAPDH
f
Control
POC1B-/-
POC1A/ POC1B-/-
70 kDa
55 kDa
37 kDa
POC1B
GAPDH
g
POC1A-/-
12.8 kb
Gene (79467 bp)
5000 bp
Exon 2
Exon 7
Protein (407 aa)
WD40
CC
aa 10
301
358
405
407
h
KO allele 1
Exon 2
Exon 7
1 bp insertion + large deletion
KO allele 2
Intron 1
Exon 7
large deletion
wild type allele
Exon 3
i
POC1B-/-
12.7 kb
Gene (106330 bp)
5000 bp
Intron 4
Exon 10
Protein (478 aa)
WD40
CC
aa 10
301
426
475
478
j
KO allele
Intron 4
Exon 10
large deletion
wild type allele
Intron 9
Exon 10

**Supplementary Figure 2. Generation of *POC1* knockout cell lines in RPE1 tetON *TP53*$^{-/-}$ using a CRISPR/Cas9 dual sgRNA strategy**

**a** Localisation of the epitopes recognised by the used antibodies in the respective proteins. For FAM161A, MDM1 and CEP44 the MT-binding site is indicated. **b** IF analysis of the *POC1A*$^{-/-}$ and *POC1B*$^{-/-}$ cell lines using antibodies against POC1A or POC1B (green) and γ-tubulin (red). Scale bars: 10 µm, magnification scale bars: 1 µm. **c, d** Quantification of the normalised POC1A and POC1B signal in the knockout cell lines shown in **b**. Data are presented as mean ± SD. Statistics were derived from two-tail unpaired *t*-test analysis of N= 3 biologically independent experiments, $n$ > 150 cells per cell line for each experiment. Source data are provided as a Source Data file. **e, f** Verification of the knockout cell lines by immunoblotting (IB) with the indicated antibodies. In addition, a *POC1A/B*$^{-/-}$ double knockout cell line was included. GAPDH was used as a loading control. **g** Scheme of the *POC1A* WT gene and the domain architecture of the protein. The sgRNAs used to generate the *POC1A* knockout are shown. **h** Chromatogram of the sequenced knockout clone showing successful deletion. Allele 1 shows in exon 2 an insertion of 1 bp following a large deletion, after which the sequence of exon 7 follows. Allele 2 has a large deletion starting already in intron 1, after which the sequence of exon 7 is following. In comparison, in the WT allele of control cells, the sequence of exon 3 can be amplified with PCR and subsequently sequenced. **i** Scheme of the *POC1B* WT gene, domain architecture of the protein and the sgRNAs used to generate the *POC1B* knockout. **j** Chromatogram of the sequenced knockout clone showing a successful deletion. The clone shows a homozygous deletion, in which both alleles have a large deletion in intron 4, after which the sequence of exon 10 is following. In comparison, the WT allele of control cells, the border between intron 9 and exon 10 is shown.

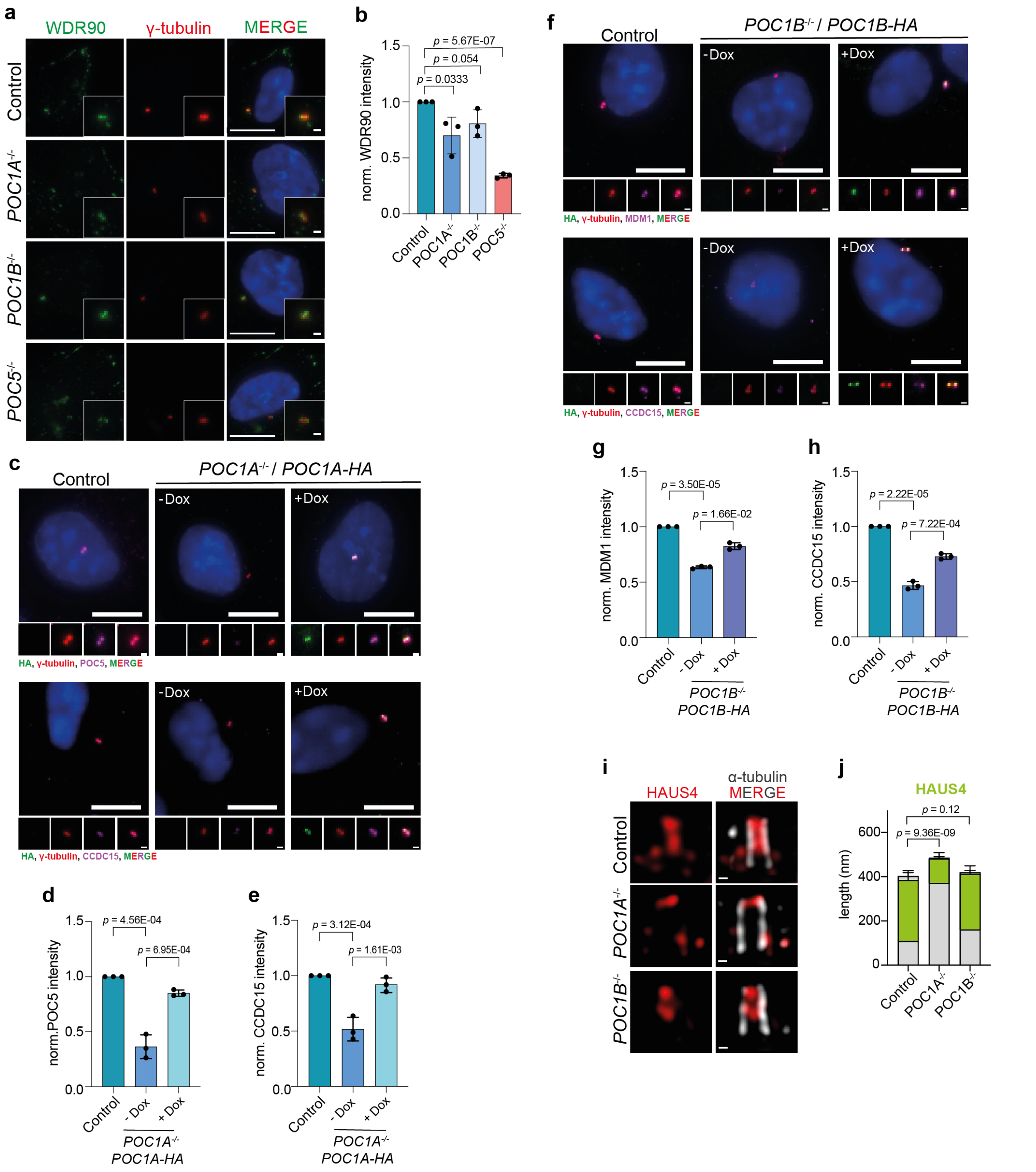


**Supplementary Figure 3. POC1 proteins are important for localisation of inner luminal centriole proteins**
**a** IF images of control, *POC1A*$^{-/-}$, *POC1B*$^{-/-}$ and *POC5*$^{-/-}$ cell lines stained for WDR90 (green) and γ-tubulin (red). Scale bars: 10 µm, magnification scale bars: 1 µm. **b** Quantification of the signal intensities shown in **a**. Data are presented as mean ± SD. Statistics were derived from two-tail unpaired *t*-test analysis of N= 3 biologically independent experiments, *n* > 100 cells per cell line for each experiment. **c** IF images of *POC1A*$^{-/-}$ cells complemented with (+) and without (-) a Dox-inducible HA-tagged *POC1A* construct (green) to test if inner scaffold proteins POC5 and CCDC15 (magenta) can be recruited to centrioles. **d, e** Quantification of **c**. Signal intensities of POC5 and CCDC15 at the centrosome were quantified. **f** *POC1B*$^{-/-}$ cells was complemented with (+) and without (-) a Dox-inducible HA-tagged *POC1B* construct and stained against the respective proteins MDM1 and CCDC15. **g, h** Quantification of **f**. Signal intensities of MDM1 and CCDC15 at the centrosome were quantified. **c, f** Scale bars: 5 µm, magnification scale bars: 1 µm. **d, e, g, h** Data are presented as mean ± SD. Statistics were derived from two-tail unpaired *t*-test analysis of N= 3 biologically independent experiments, *n* > 100 cells per cell line for each experiment. **i** HAUS4, a subunit of the augmin complex[13], was stained in the two *POC1*$^{-/-}$ cell lines and analysed by U-ExM. Scale bars: 100 nm. **j** Quantification of the signal length of HAUS4 shown in **i**. Data are presented as mean ± SD. All statistics were derived from two-tail unpaired *t*-test analysis. *n*= 9 (Control), 8 (*POC1A*$^{-/-}$), 8 (*POC1B*$^{-/-}$) centrioles. **b**, **d**, **e**, **g**, **h** an **j** Source data are provided as a Source Data file.

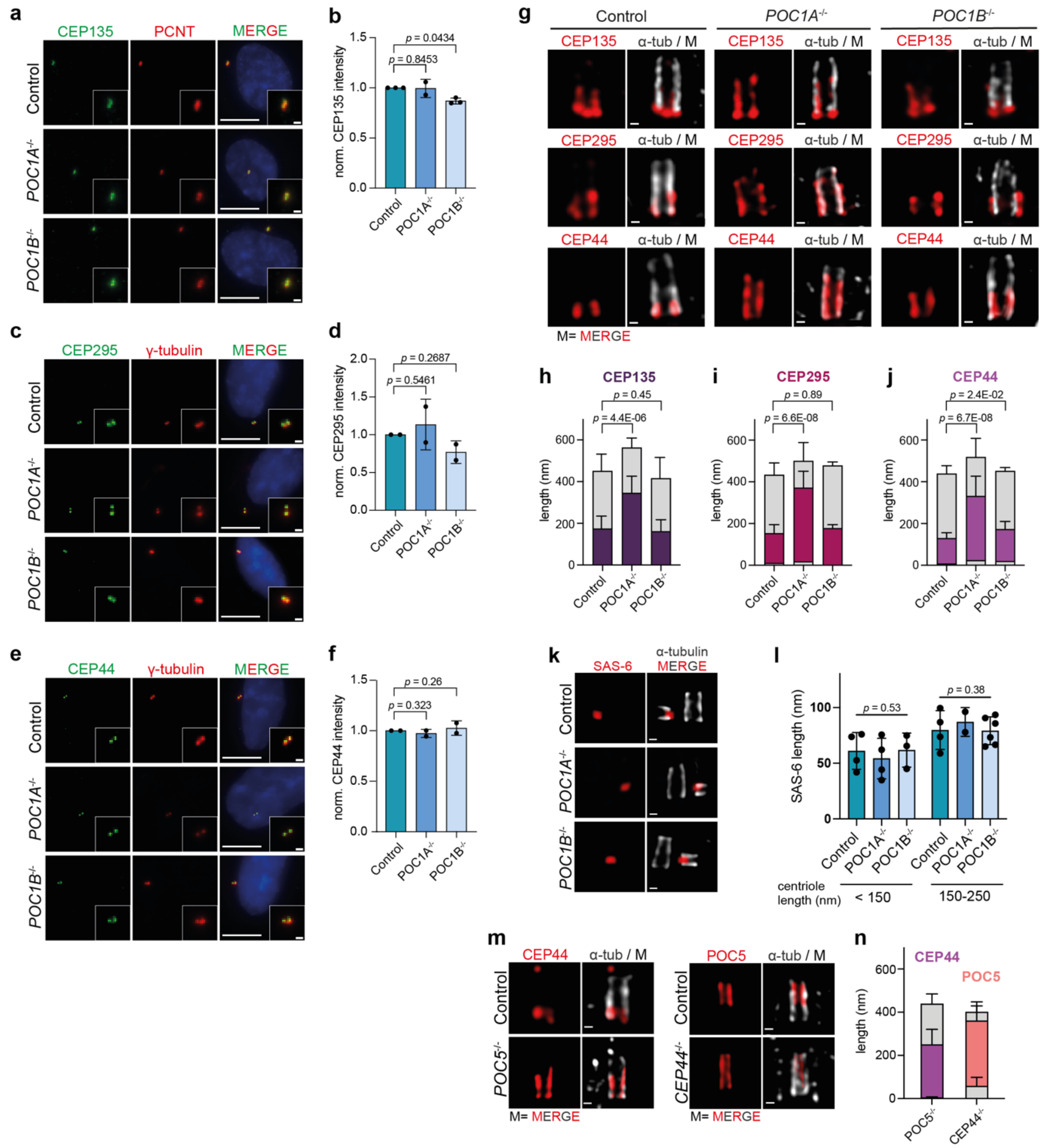


**Supplementary Figure 4. Centriole substructures can influence the distribution of centriolar proteins**

**a, c, e** IF images of the proximal proteins CEP135, CEP295 and CEP44 in *POC1A*$^{-/-}$ and *POC1B*$^{-/-}$ cell lines. Scale bars: 10 µm, magnification scale bars: 1 µm. **b, d, f** Quantification of **a, c, e**. Data are presented as mean ± SD. Statistics were derived from two-tail unpaired *t*-test of N= 3 biologically independent experiments, *n* > 100 cells per cell line for each experiment for CEP135 in Control and *POC1B*$^{-/-}$ cells and N=2 biologically independent experiments, *n* > 100 cells per cell line for each experiment for CEP295 and CEP44; and CEP135 in *POC1A*$^{-/-}$. **g** U-ExM images of centrioles from control and knockout cell lines stained against α-tubulin (grey) and the indicated proteins (red), M= merged channels. **h-j** Quantification of **g**. The signal distribution of the respective proteins in each cell line along the centrioles was measured. Signals extending out of centrioles (proximal) were not included in the quantification. Data are presented as mean ± SD. All statistics were derived from two-tail unpaired *t*-test. **h** *n*= 16 (Control), 10 (*POC1A*$^{-/-}$), 11 (*POC1B*$^{-/-}$) centrioles. **i** *n*= 12 (Control), 11 (*POC1A*$^{-/-}$), 10 (*POC1B*$^{-/-}$) centrioles. **j** *n*= 14 (Control), 18 (*POC1A*$^{-/-}$), 10 (*POC1B*$^{-/-}$) centrioles. **k** U-ExM images of the *POC1*$^{-/-}$ cell lines stained against SAS-6 (red) and α-tubulin (grey). **l** Quantification of **k**. Procentrioles were grouped based on their length of the α-tubulin signal and the length of the SAS-6 signal was measured to prevent artificial differences due to different procentriole length. Statistics were derived from one-way ANOVA. **m** CEP44 (red) was stained in a control and *POC5*$^{-/-}$ cell line. Additionally, POC5 (red) was stained in a control and *CEP44*$^{-/-}$ cell line. α-tubulin (grey), M: merged channels. **n** Quantification of **m**. The length of CEP44 as well as POC5 is changed in the respective knockout cell lines. The error bars represent the SD. *n*= 9 (*POC5*$^{-/-}$) and 15 (*CEP44*$^{-/-}$) centrioles. **g, k, m** Scale bars: 100 nm. **b, d, f, h, i, j, l** and **n** Source data are provided as a Source Data file.

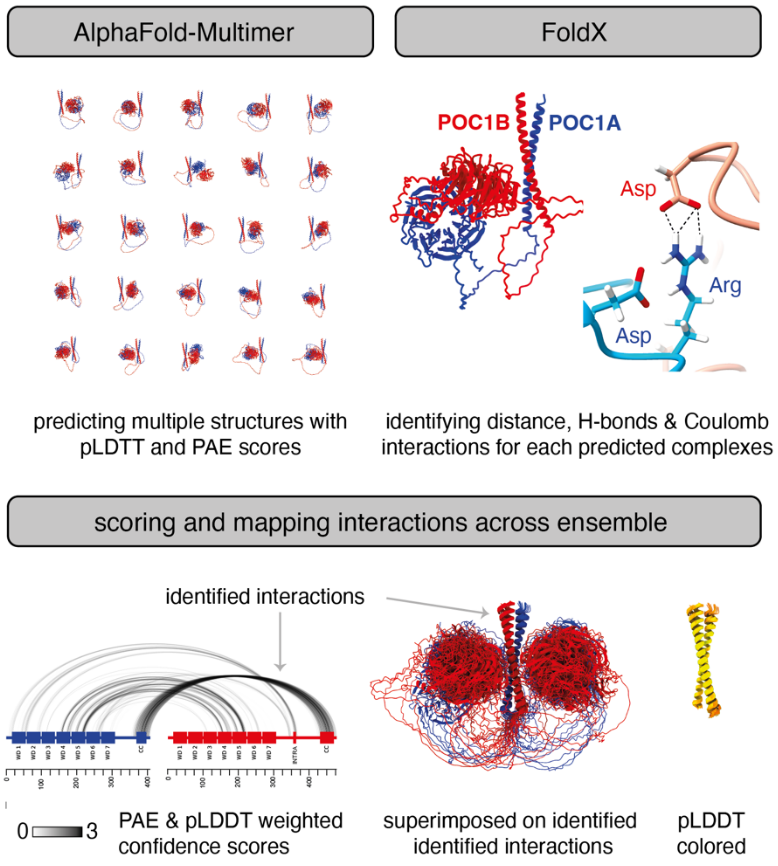


**Supplementary Figure 5. Pipeline for establishing ensemble interactions maps**
Using AlphaFold-Multimer (an extension of AlphaFold2), multiple prediction runs for each potential protein-protein interaction were performed, providing multiple structures with their corresponding pLDTT score and PAE plots. FoldX, an algorithm used for determining energy differences for example upon mutagenesis, identified distances, H-bonds and Couloumb interactions for each predicted protein complexes. Together with PAE and plDDT weighted confidence scores, potential interactions were mapped and scored across the ensembles and could be identified.

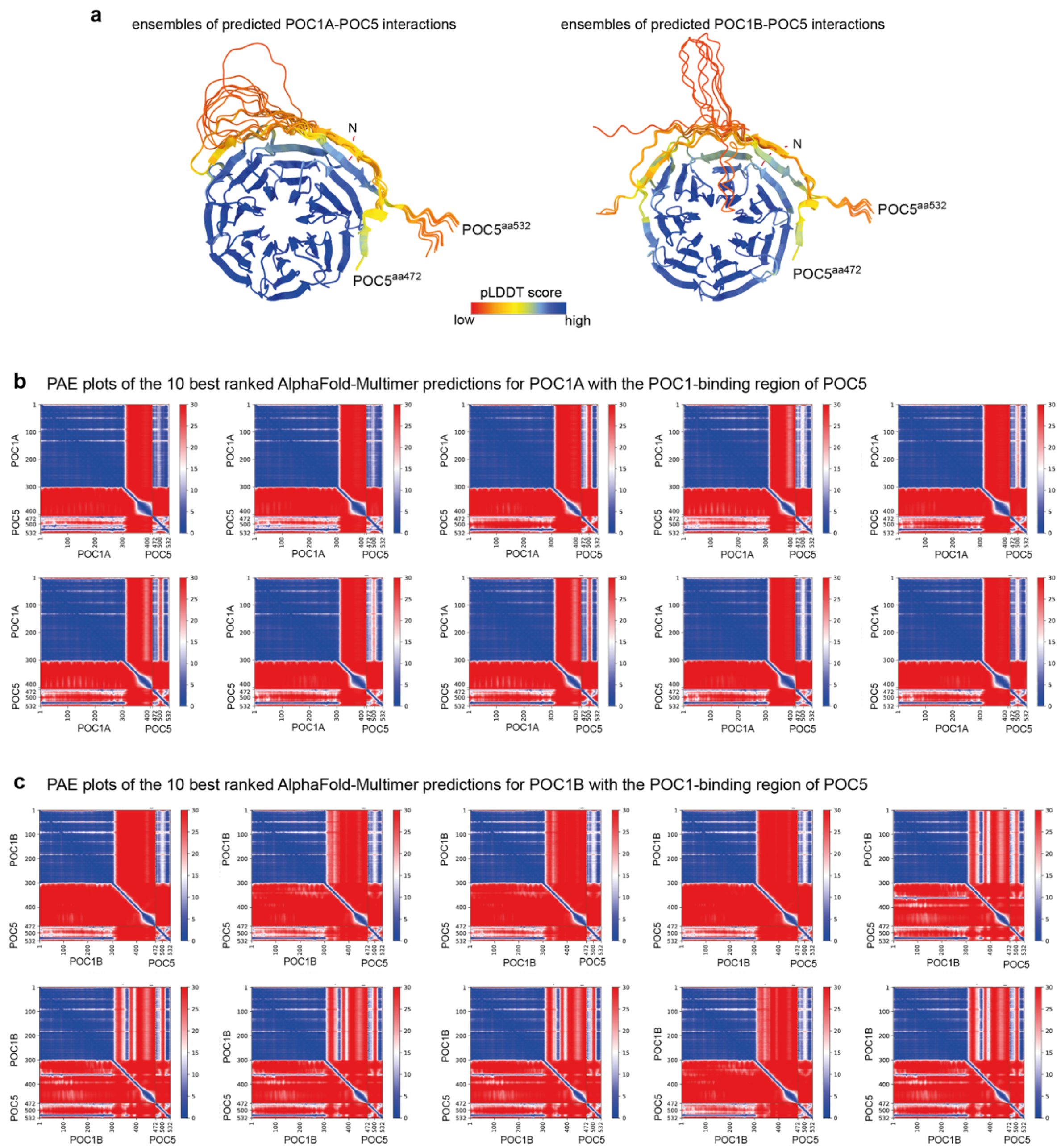


**Supplementary Figure 6. The WD40 domain of POC1 is important for the interaction with POC5**

**a** Ensembles of the 10 best ranked AlphaFold-Multimer predictions showing an interaction between the POC1 proteins and residues 472-532 of POC5. For the POC1 proteins, only the WD40 domain is displayed; additionally, the intra region is shown for POC1B. Colouring is based on pLDDT score. **c**, **d** PAE plots of the 10 best ranked AlphaFold-Multimer predictions for interactions between the POC1 proteins and the POC1-binding region of POC5 (residues 472-532).

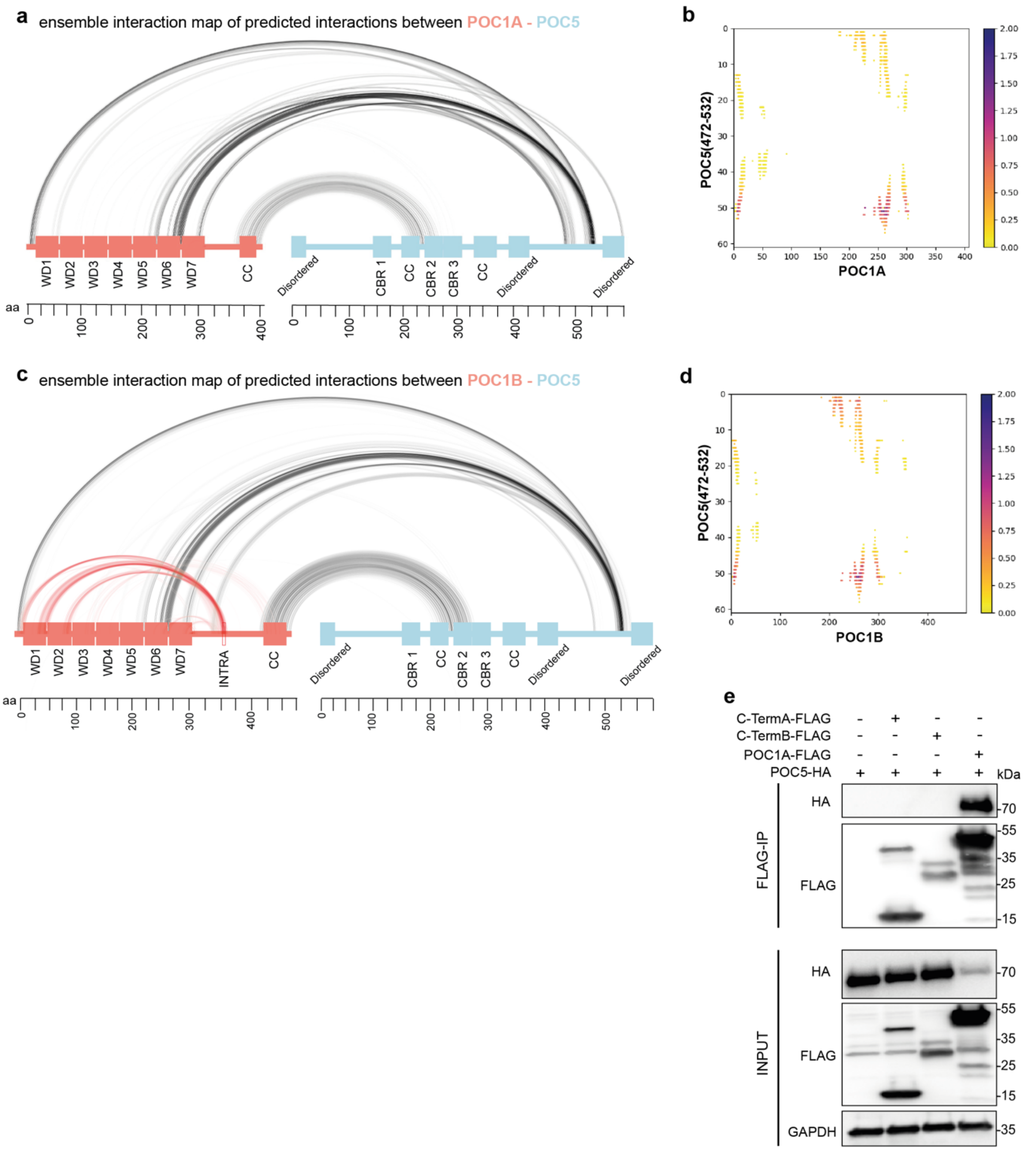


**Supplementary Figure 7. POC5 interacts with POC1 through a binding region in its C-terminus**

**a** Ensemble interaction map based on AlphaFold-Multimer predictions of an interaction between POC1A and full-length POC5. The most robust interactions are predicted to be between the WD40 domain of POC1A and the C-terminus of POC5. **b** Plotted scores of the interaction map shown in **a** with a focus on POC1A full-length and the residues 472-532 of POC5. Higher score correlates with more robust predicted interaction. **c** Ensemble interaction map based on AlphaFold-Multimer predictions of an interaction between POC1B and full-length POC5. Note that the WD40 domain is predicted to interact with the Intra region of POC1B. **d** Plotted scores of the interaction map shown in **c** with a focus on POC1B full-length and the residues 472-532 of POC5. Higher score correlates with more robust predicted interaction. **e** Representative FLAG IP from HEK293T cells expressing the C-terminal region consisting of the linker and the coiled-coil region of the POC1 proteins tagged with FLAG together with HA-tagged POC5. Note, although POC5-HA in lane 4 was only weakly expressed, the IP with POC1A (HA blot) was very efficient. The C-terminus of POC1 cannot immunoprecipitate POC5, verifying the interacting site in the WD40 domain. GAPDH is used as input control.

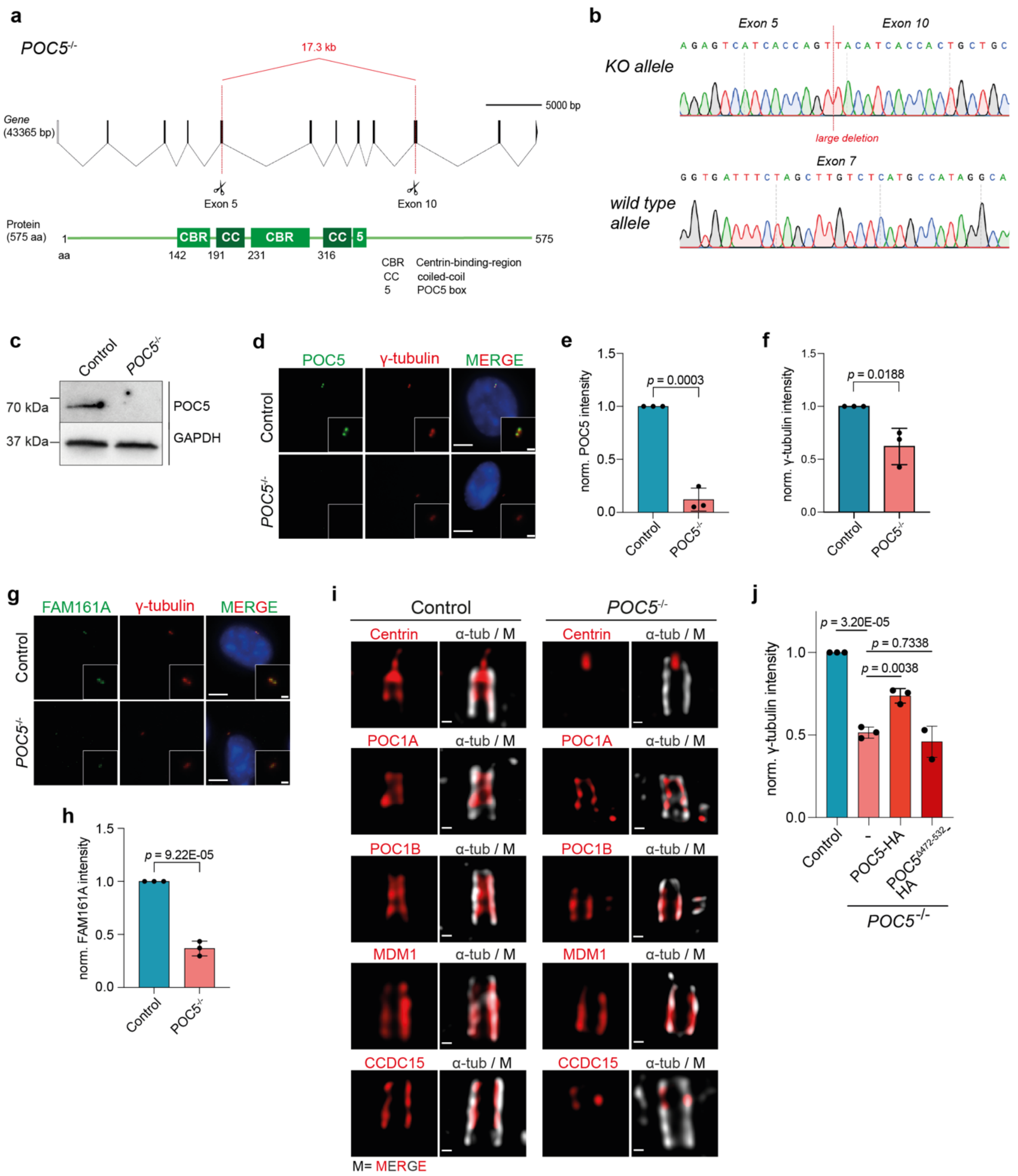


**Supplementary Figure 8. Generation of *POC5* knockout cell line in RPE1 tetON *TP53*$^{-/-}$ using a CRISPR/Cas9 dual sgRNA strategy**

**a** Scheme of the *POC5* WT gene and the domain architecture of the protein. The sgRNAs used to generate the knockout targeting exon 5 and exon 10 are shown, resulting in a deletion of 17.3 kb. **b** Chromatogram of the sequenced knockout clone showing successful deletion. The clone shows a homozygous deletion, in which both alleles have the large deletion in exon 5, after which the sequence of exon 10 is following. In comparison, in the *POC5* WT allele of control cells, the sequence of exon 7 can be amplified with PCR and subsequently sequenced. **c** Verification of the knockout cell line by IB. GAPDH was used as a loading control. **d, g** IF images of the *POC5*$^{-/-}$ cell line stained against the indicated proteins verifying the loss of POC5 at the centrosomes. Scale bars: 5 µm, magnification scale bars: 1 µm. **e, f, h** Quantification of the signal intensities from **d** and **g. i** U-ExM of *POC5*$^{-/-}$ centrioles with near-normal length. The samples were stained against the indicated antibodies (red) and α-tubulin (grey). Like in the *POC1A*$^{-/-}$, the middle pool of Centrin is lost in *POC5*$^{-/-}$ cells. Localisation of POC1A, POC1B and MDM1 is unaffected in *POC5*$^{-/-}$ cells. Similar to the *POC1* knockouts, CCDC15 is also affected in *POC5*$^{-/-}$ cells. Scale bar: 100 nm. **j** Complementation of *POC5*$^{-/-}$ cells with WT *POC5* can restore γ-tubulin signal at centrosomes. In contrast, expression of *POC5*$^{\Delta 475\text{-}532}$ lacking the POC1 binding site is insufficient for γ-tubulin recruitment. Images are shown in Fig. 4a. **e, f, h** and **j** Data are presented as mean ± SD. All statistics were derived from two-tail unpaired *t*-test analysis of N= 3 biologically independent experiments, *n* > 100 cells per cell line for each experiment. Source data are provided as a Source Data file.

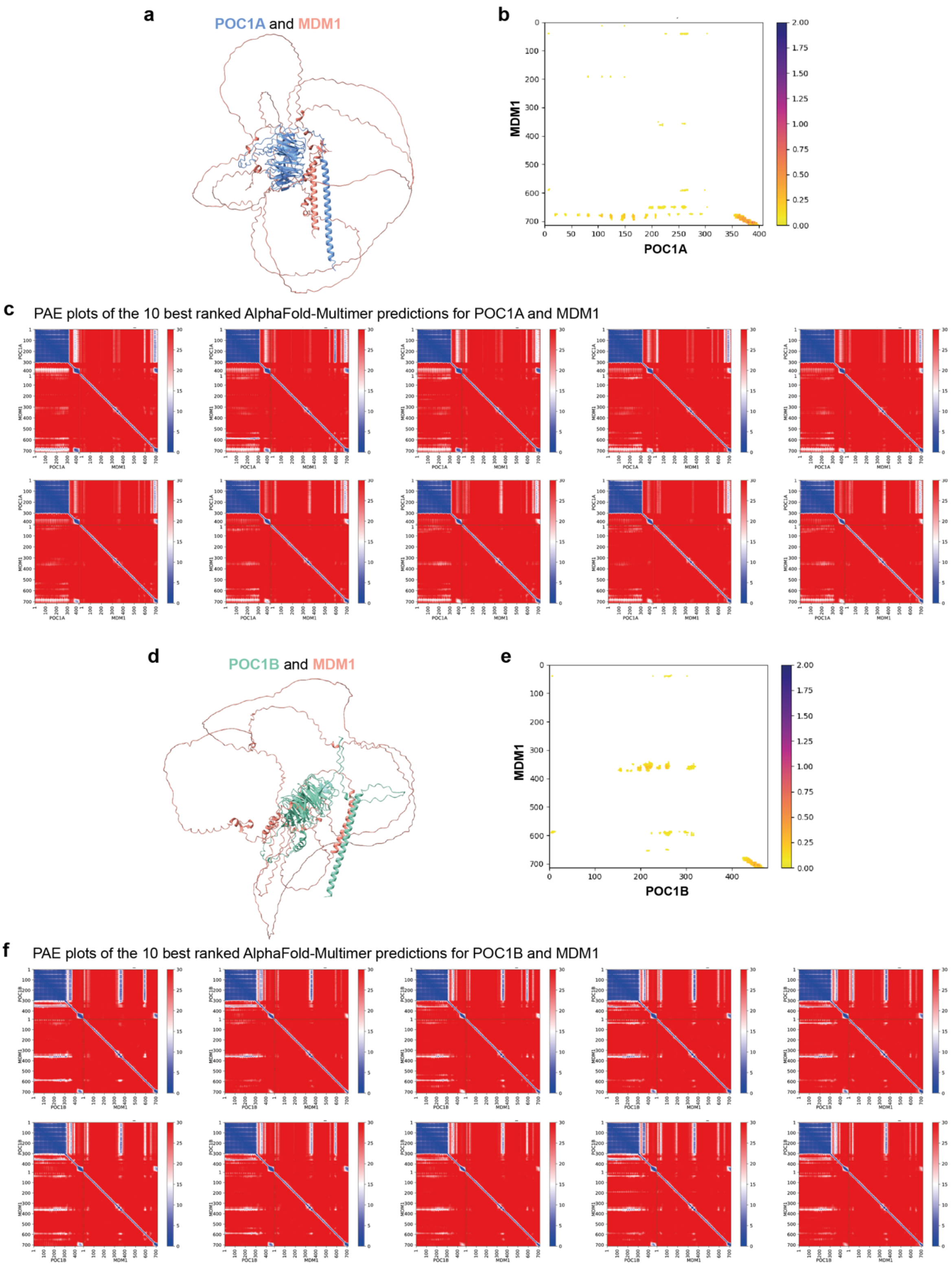


**Supplementary Figure 9. Predicted AlphaFold-Multimer interactions between MDM1 and the POC1 proteins**
**a, d** AlphaFold-Multimer predictions of the interactions between POC1A (blue) or POC1B (green) and MDM1 (salmon) showing an interaction between the C-termini of both proteins. **b, e** Plotted scores of the interaction maps of the predicted interactions between POC1A-MDM1 and POC1B-MDM1 shown in Fig. 4e. **c, f** PAE plots of the 10 best ranked predictions for the interactions between the POC1 proteins and MDM1.

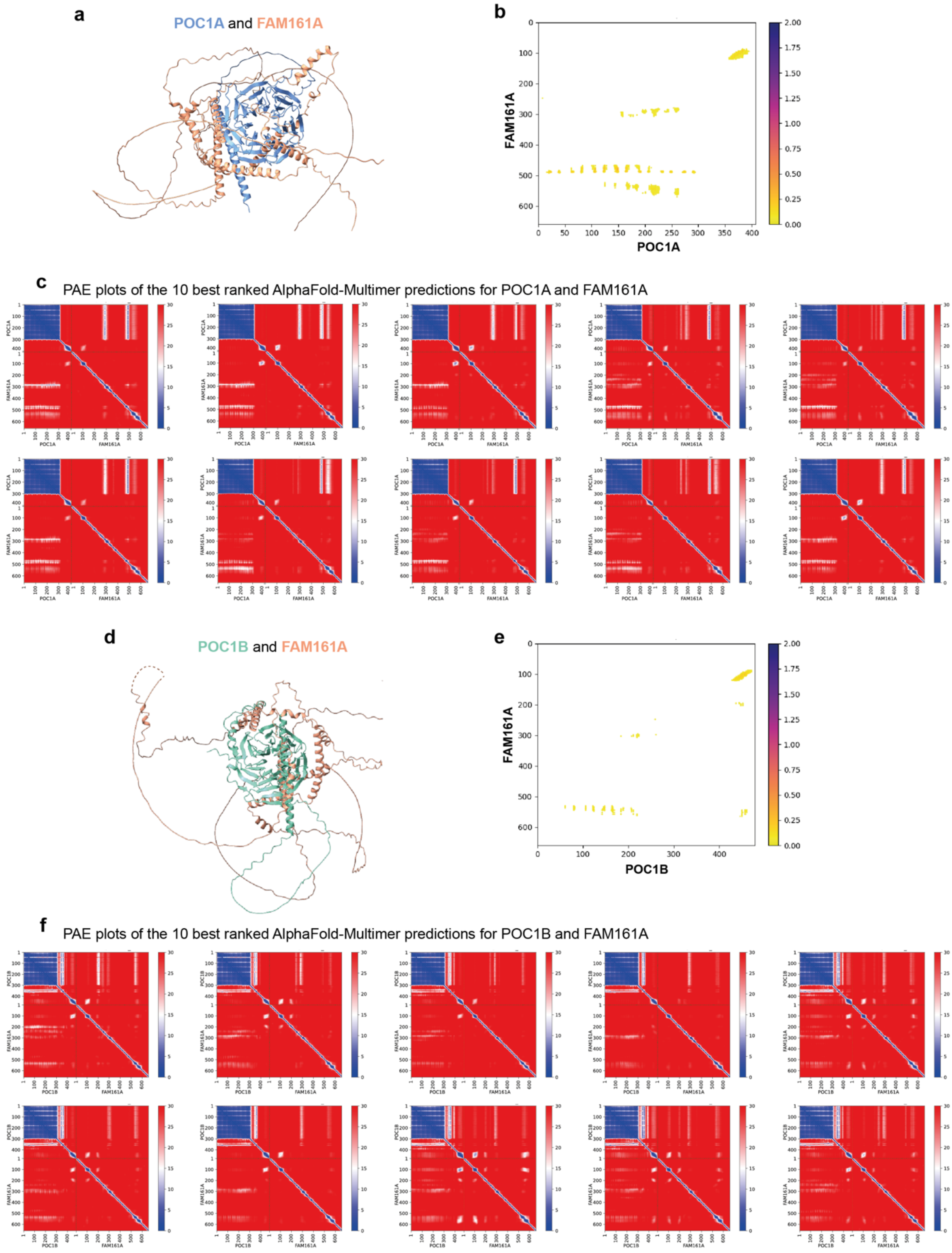


**Supplementary Figure 10. Predicted AlphaFold-Multimer interactions between FAM161A and the POC1 proteins**
**a, d** AlphaFold-Multimer predictions of the interactions between POC1A (blue) or POC1B (green) and FAM161A (salmon). The WD40 domains as well as the coiled-coil region of the POC1 proteins might be involved in the interactions. **b, e** Plotted scores of the interaction maps of the predicted interactions between POC1A-FAM161A and POC1B-FAM161A shown in Fig. 4g. **c, f** PAE plots of the 10 best ranked predictions for the interactions between the POC1 proteins and FAM161A.

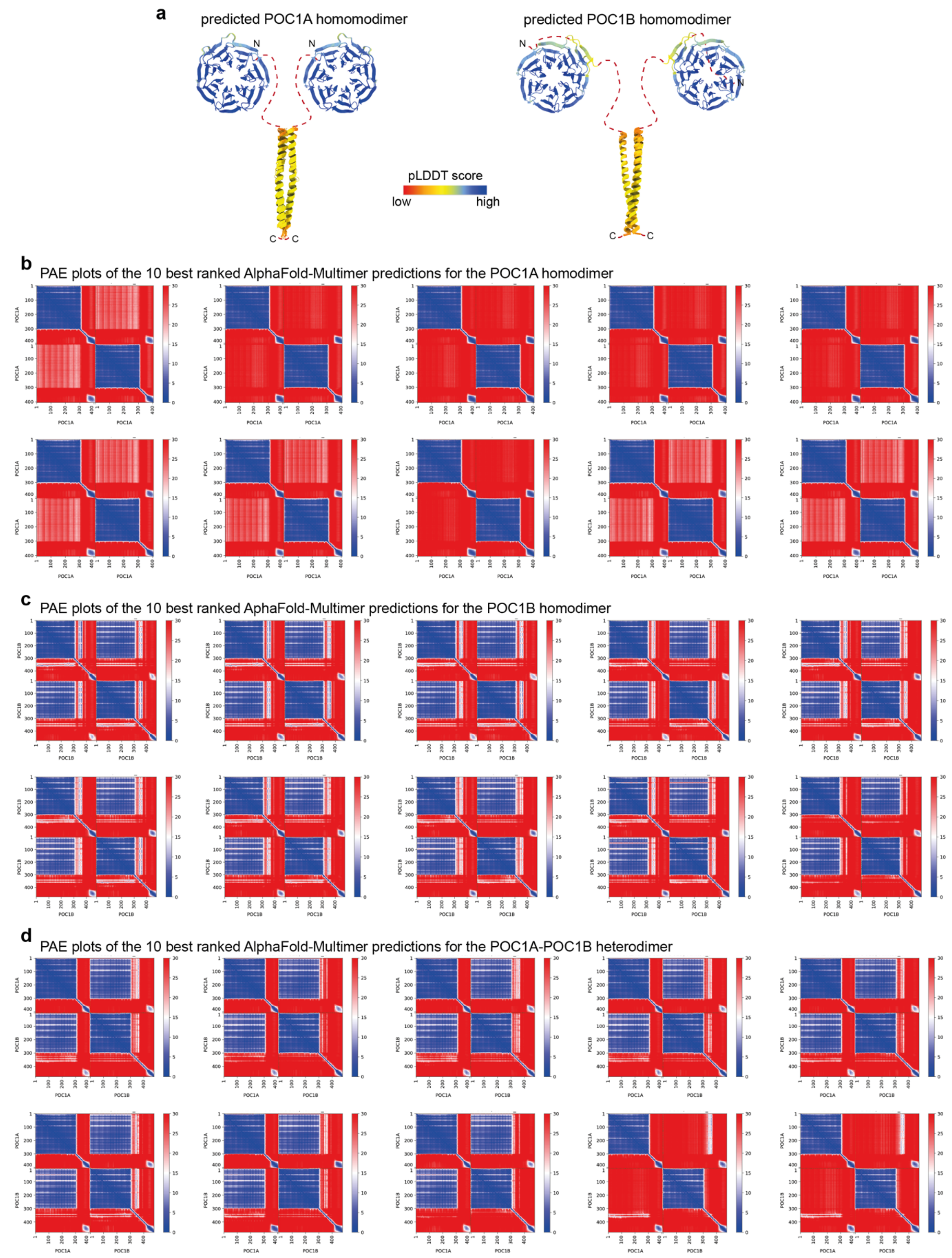


**Supplementary Figure 11. POC1 homo- and heterodimerization is mediated by the C-terminal coiled-coil region**
**a** Ensembles of the 10 best ranked AlphaFold-Multimer predictions showing the formation of POC1A and POC1B homodimers that are mediated by the C-terminal coiled-coil regions. Colouring based on pLDDT score. **b-d** PAE plots of the 10 best ranked predicted POC1 homo- and heterodimers.

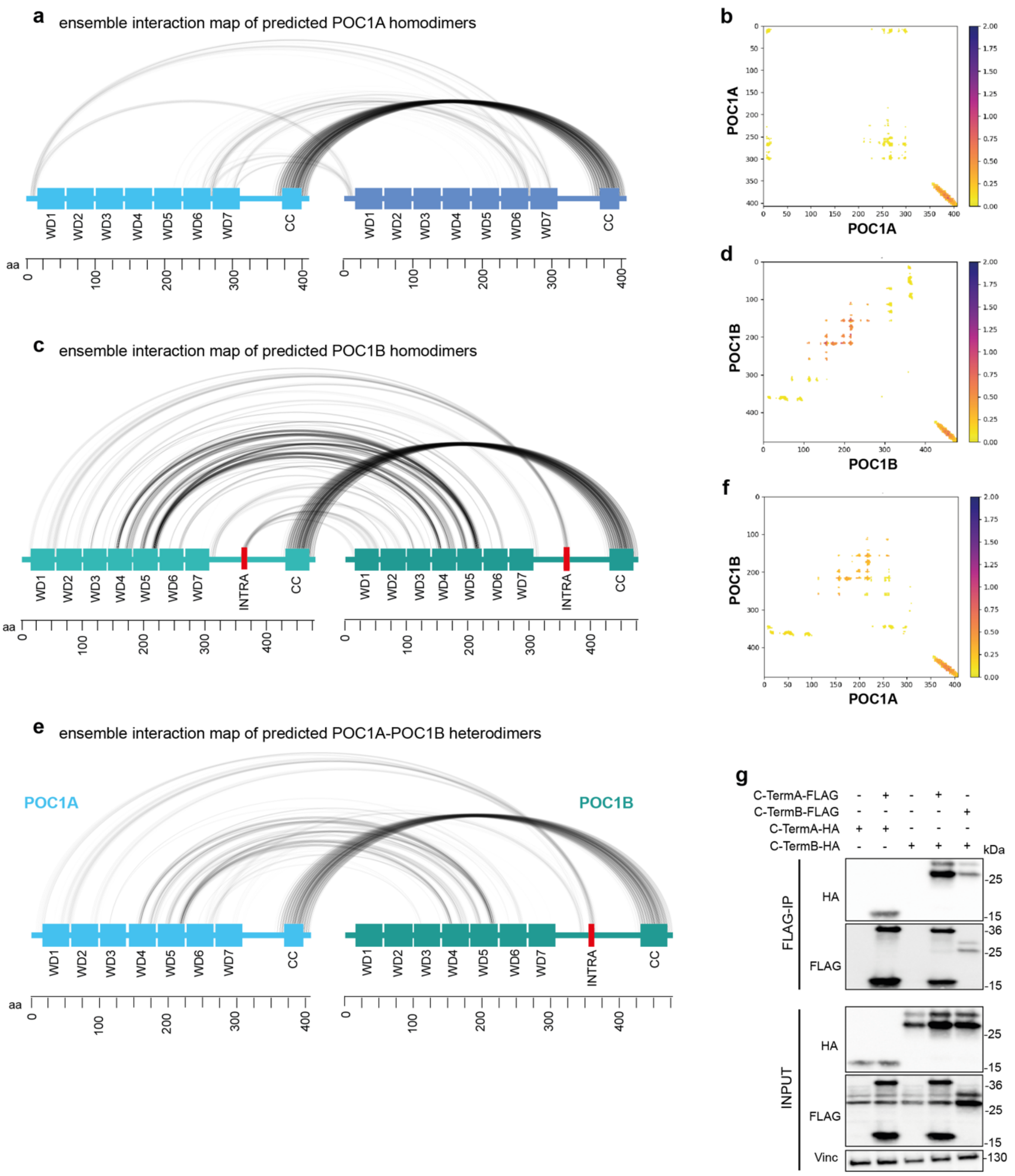

**Supplementary Figure 12. POC1A and POC1B form homo- and heterodimers via their coiled-coil regions**
**a, c, e** Ensemble interaction maps based on AlphaFold-Multimer predictions between POC1A homodimer (**a**), POC1B homodimer (**c**) or POC1A-POC1B heterodimer (**e**). The interaction between homo- and heterodimers is mainly mediated by the C-terminal coil-coiled region of the POC1 proteins. **b, d, f** Corresponding score plots for the interaction maps of POC1A or POC1B homodimers and POC1A-POC1B heterodimer. **g** FLAG-IP of HEK293 cells expressing FLAG and HA-tagged C-terminal domains of POC1A or POC1B to verify that interactions are mediated by the C-terminus. Vinculin was used as a loading control.

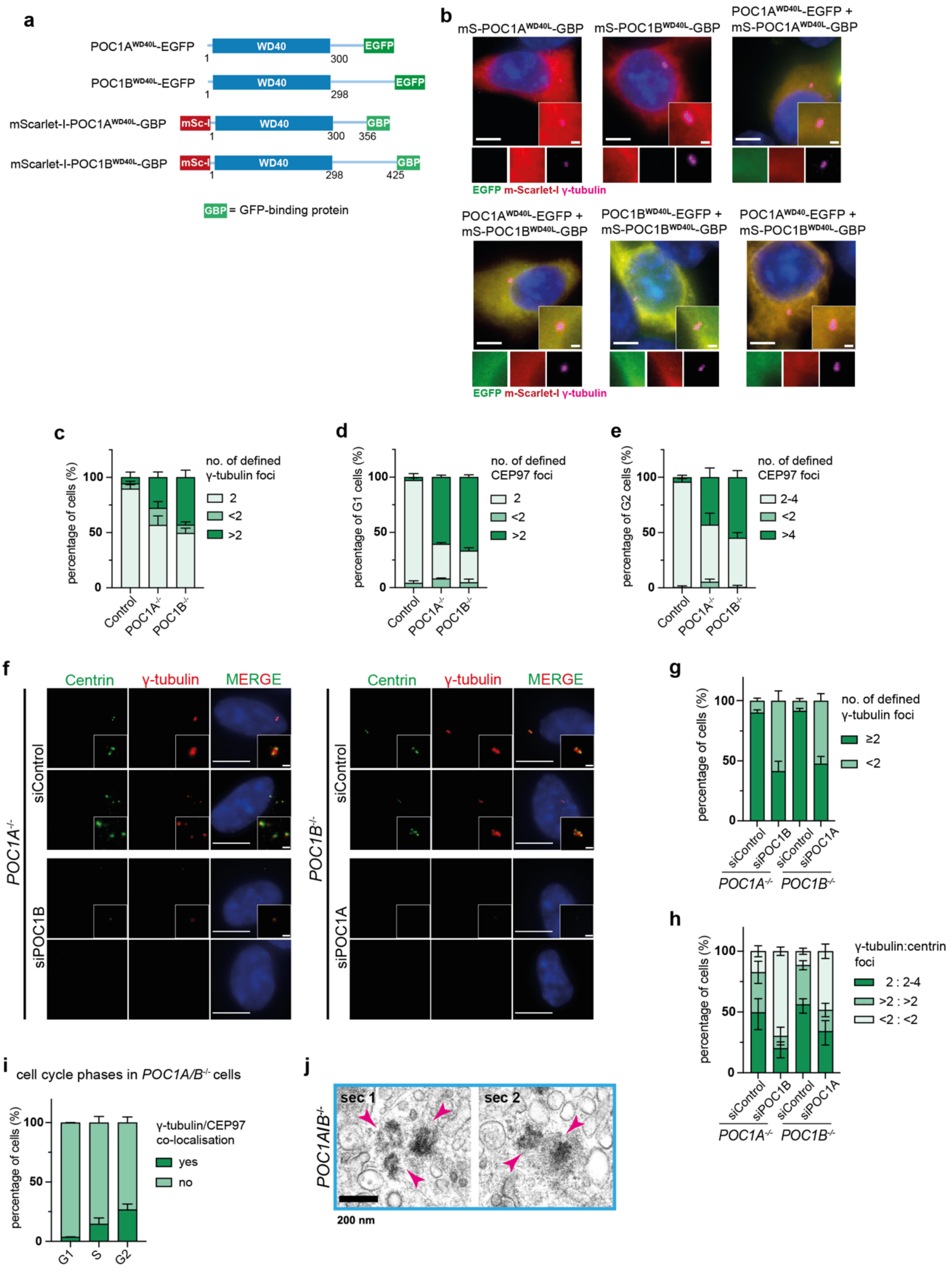
a
POC1AWD40L-EGFP
WD40
EGFP
1
300
POC1BWD40L-EGFP
298
mScarlet-I-POC1AWD40L-GBP
mSc-I
GBP
356
mScarlet-I-POC1BWD40L-GBP
425
GBP = GFP-binding protein
b
mS-POC1AWD40L-GBP
mS-POC1BWD40L-GBP
POC1AWD40L-EGFP + mS-POC1AWD40L-GBP
EGFP m-Scarlet-I γ-tubulin
POC1AWD40L-EGFP + mS-POC1BWD40L-GBP
POC1BWD40L-EGFP + mS-POC1BWD40L-GBP
POC1AWD40-EGFP + mS-POC1BWD40L-GBP
c
percentage of cells (%)
no. of defined γ-tubulin foci
2
<2
>2
Control
POC1A-/-
POC1B-/-
d
percentage of G1 cells (%)
no. of defined CEP97 foci
e
percentage of G2 cells (%)
2-4
>4
f
Centrin
γ-tubulin
MERGE
POC1A-/-
siControl
siPOC1B
POC1B-/-
siPOC1A
g
no. of defined γ-tubulin foci
≥2
h
γ-tubulin:centrin foci
2 : 2-4
>2 : >2
<2 : <2
i
cell cycle phases in POC1A/B-/- cells
γ-tubulin/CEP97 co-localisation
yes
no
G1
S
G2
j
POC1A/B-/-
sec 1
sec 2
200 nm

**Supplementary Figure 13. siRNA depletion of POC1A or POC1B in the POC1 knockout cell lines mimic the phenotype of the *POC1A/B$^{-/-}$* double knockout**

**a** Additional constructs used for the dimerization of POC1 subdomains with GFP/GBP. The constructs contain the WD40 domain and the flexible linker region between the WD40 and coil-coiled region (hence, WD40L). **b** IF images of HEK293 cells expressing the constructs shown in **a**. The WD40 domains do not show a specific centrosomal localisation, regardless of having the linker region included. See Fig. 5f-g for additional constructs. EGFP (green), m-Scarlet-I (red), γ-tubulin (magenta). Scale bars: 5 µm, magnification scale bars: 1 µm. **c** Interphase *POC1$^{-/-}$* cells show an increase in the number of γ-tubulin foci, indicating centrosome amplification. **d, e** Centriole numbers in G1 and G2 cells marked by CEP97 signal. Centriole numbers are altered in the *POC1$^{-/-}$* knockout cell lines, leading to an increase of overamplified centrioles in G2 phase. **f** IF images of *POC1$^{-/-}$* cells treated with siRNAs against *POC1A* and *POC1B* and stained against Centrin (green) and γ-tubulin (red). Scale bars: 10 µm, magnification scale bars: 1 µm. **g, h** Quantification of the Centrin and γ-tubulin foci shown in **f**. After siRNA depletion, centriole and centrosome numbers decrease. **i** Quantification of CEP97 and γ-tubulin co-localisation in the specific cell cycle phases of *POC1A/B$^{-/-}$* cells. During S and G2 phase, the percentage of cells showing co-localisation is increasing. Data are presented as mean ± SD. *N*=3 biologically independent experiments, n > 80 cells per cell cycle phase and per cell line in each experiment. **c-e** and **g-h** Data are presented as mean ± SD. *N*=2 biologically independent experiments, n > 100 cells per cell line for each experiment, except for **h,** where *N*=3, n > 70 cells per cell line. Source data are provided as a Source Data file. **j** EM images of *POC1A/B$^{-/-}$* cells. In most *POC1A/B$^{-/-}$* cells, no clear centriolar structure was observed. In rare cases, centriole fragments were detected by EM. Scale bar: 200 nm.

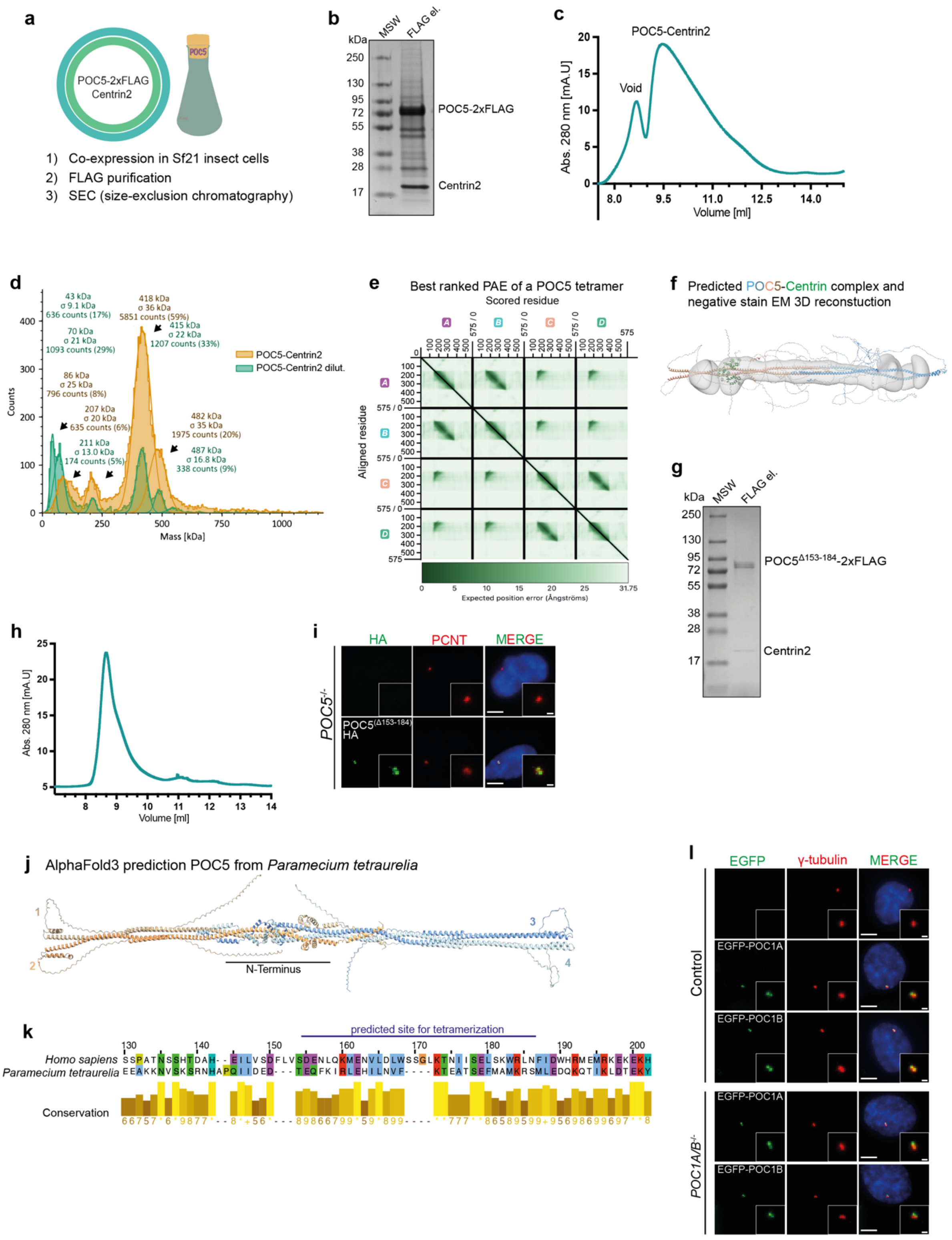
a
POC5-2xFLAG
Centrin2
POC5
1) Co-expression in Sf21 insect cells
2) FLAG purification
3) SEC (size-exclusion chromatography)
b
kDa
MSW
FLAG el.
250
130
95
72
55
38
28
17
POC5-2xFLAG
Centrin2
c
POC5-Centrin2
Void
Abs. 280 nm [mA.U]
Volume [ml]
d
43 kDa
σ 9.1 kDa
636 counts (17%)
418 kDa
σ 36 kDa
5851 counts (59%)
415 kDa
σ 22 kDa
1207 counts (33%)
70 kDa
σ 21 kDa
1093 counts (29%)
86 kDa
σ 25 kDa
796 counts (8%)
207 kDa
σ 20 kDa
635 counts (6%)
211 kDa
σ 13.0 kDa
174 counts (5%)
482 kDa
σ 35 kDa
1975 counts (20%)
487 kDa
σ 16.8 kDa
338 counts (9%)
POC5-Centrin2
POC5-Centrin2 dilut.
Counts
Mass [kDa]
e
Best ranked PAE of a POC5 tetramer
Scored residue
Aligned residue
Expected position error (Ångströms)
f
Predicted POC5-Centrin complex and
negative stain EM 3D reconstuction
g
kDa
MSW
FLAG el.
POC5Δ153-184-2xFLAG
Centrin2
h
Abs. 280 nm [mA U]
Volume [ml]
i
HA
PCNT
MERGE
POC5-/-
POC5(Δ153-184)
HA
j
AlphaFold3 prediction POC5 from Paramecium tetraurelia
N-Terminus
k
predicted site for tetramerization
Homo sapiens
Paramecium tetraurelia
Conservation
l
EGFP
γ-tubulin
MERGE
Control
EGFP-POC1A
EGFP-POC1B
POC1A/B-/-

**Supplementary Figure 14. Purification and characterization of the human POC5-centrin complex from insect cells**
**a** Experimental setup for expressing human genes in insect cells for purification. Baculovirus was used to express the construct in Sf21 insect cells and after harvesting and lysis, FLAG purification was performed. FLAG-eluates were loaded on a size exclusion chromatography (SEC) column. **b** Coomassie Blue stained SDS-PAGE of the POC5-Centrin2 FLAG elution. **c** Chromatogram of the SEC purification (using Superdex 6 increase column) for the POC5-Centrin2 construct. N= 3 biologically independent experiments for protein expression and purification. **d** Mass photometry histogram of the purified POC5 sample shown in **c**. A distinct peak can be observed at 415-418 kDa for the undiluted (orange) and diluted (green) sample corresponding to the tetrameric formation of POC5 bound to multiple Centrin2 molecules. Experiment performed once with n= 2 measurements. **e** Top ranked PAE of the predicted POC5 tetramer. The PAE plot was visualised using PAE Viewer[14]. **f** Superimposed image of the AlphaFold-Multimer predicted structure of the POC5-Centrin2 complex with four POC5 and two Centrin2 molecules and the experimentally observed negative stain EM 3D reconstructed structure from single particle averaging. Overlay for visualisation purposes. **g** Coomassie Blue stained SDS-PAGE of the POC5$^{\Delta 153\text{-}184}$ -Centrin2 FLAG elution. **h** Chromatogram of SEC purification (using Superdex 75 column) for the POC5$^{\Delta 153\text{-}184}$-Centrin2 construct. N= 2 biologically independent experiments for protein expression and purification. **i** *POC5*$^{-/-}$ cells without *POC5* construct (top) or expressing HA-tagged *POC5*$^{\Delta 153\text{-}184}$ that lacks the site for tetramerization (bottom). The POC5$^{\Delta 153\text{-}184}$ mutant can still localise to centrosomes marked by PCNT. **j** AlphaFold3[15] prediction of POC5 from *Paramecium tetraurelia*. The ability to form a tetramer occurs also in *Paramecium*. **k** Alignment of the region important for tetramerization from human and *Paramecium* POC5 (UniProtKB accession number: A0BL87). This region shows high conservation. **l** N-terminally EGFP-tagged versions of POC1A or POC1B are expressed in control and *POC1A/B*$^{-/-}$ cells. Tagging of the N-terminus does not affect centrosomal localisation. **i** and **l** Scale bars: 5 µm, magnification scale bars: 1 µm.

# Supplementary Table 1: Primer list

| **Construct/ Primer** | **Sequence (5' – > 3')** |
|---|---|
| pRetrox-TRE3G-POC1A-FLAG | For: GGATCCATCGATACGCGTGCGCCACCATGGCTGCGCCCTGCGCGGAG<br>Rev: CCCGGTAGAATTCGGGCCttaCTTGTCATCGTCGTCCTTGTAGTCTGCACCAGCTCCTGCACCTG |
| pRetrox-TRE3G-POC1A-HA | For: GTCTTATACTTGGATCCATCGATAaATGGCTGCGCC<br>Rev: CCTGCACCTGCACCAGCTCCtgcggccgcTGGTGTTGCTCTCTG |
| pRetrox-TRE3G-POC1B-FLAG | For: GGATCCATCGATACGCGTGCGCCACCATGGCCTCAGCCACGGAGGAC<br>Rev: CCCGGTAGAATTCGGGCCttaCTTGTCATCGTCGTCCTTGTAGTCTGCACCAGCTCCTGCACCTG |
| pRetrox-TRE3G-POC1B-HA | For: GTCTTATACTTGGATCCATCGATAaATGGCCTCAGCCAC<br>Rev: CCTGCACCTGCACCAGCTCCtgcggccgcGCTTTTCTGTTGGACAGC |
| pRetrox-TRE3G-C-TermA-FLAG | For: CTTATACTTGGATCCATCGATAGCCACCATGATTGTTGATCATGGAGAAGTCACG<br>Rev:<br>CTACCCGGTAGAATTCGGGCCttaCTTGTCATCGTCGTCCTTGTAGTCTGCACCAGCTCCTGCACCTGCACCAGCTCCtgcggccgc<br>TGGTGTTGCTCTCTGCATG |
| pRetrox-TRE3G-C-TermA-HA | For: ACTTTTGTCTTATACTTGGATCCATCGATAGCCACCATGATTGTTGATCATGG<br>Rev: ttaAGCGTAATCTGGAACATCGTATGGGTATGCACCAGCTCCTGCACC |
| pRetrox-TRE3G-C-TermB-FLAG | For: CTTATACTTGGATCCATCGATAGCCACCATGGAATTGCATTGTAAAGGTCTTACCAAAAG<br>Rev:<br>CTACCCGGTAGAATTCGGGCCttaCTTGTCATCGTCGTCCTTGTAGTCTGCACCAGCTCCTGCACCTGCACCAGCTCCtgcggccgc<br>GCTTTTCTGTTGGACAGCAC |
| pRetrox-TRE3G-C-TermB-HA | For: CTTATACTTGGATCCATCGATAGCCACCATGGAATTGCATTGTAAAGGTCTTACCAAAAG<br>Rev:ttaAGCGTAATCTGGAACATCGTATGGGTATGCACCAGCTCCTGCACCTGCACCAGCTCCtgcggccgcGCTTTTCTGTTGGAC<br>AGCAC |
| pRetrox-TRE3G-WD40A-FLAG | For: CTTATACTTGGATCCATCGATAGCCACCATGGCTGCGCCCTG<br>Rev:<br>CTACCCGGTAGAATTCGGGCCttaCTTGTCATCGTCGTCCTTGTAGTCTGCACCAGCTCCTGCACCTGCACCAGCTCCATCAAA<br>GTTACTCTTCCAAACCATCAC |
| pRetrox-TRE3G-WD40B-FLAG | For: CTTATACTTGGATCCATCGATAGCCACCATGGCCTCAGCCACG<br>Rev:<br>CTACCCGGTAGAATTCGGGCCttaCTTGTCATCGTCGTCCTTGTAGTCTGCACCAGCTCCTGCACCTGCACCAGCTCCATCAAA<br>GTTAGTCCTCCATAATAAGACCTG |
| pRetrox-TRE3G-WD40A-HA | For: CTTATACTTGGATCCATCGATAGCCACCATGGCTGCGCCCTG<br>Rev: ttaAGCGTAATCTGGAACATCGTATGGGTATGCACCAGCTCCTGCACC |
| pRetrox-TRE3G-WD40B-HA | for CGTGGCTGAGGCCATGGTGGCTATCGATGGATCCAAGTATAAG<br>Rev: ttaAGCGTAATCTGGAACATCGTATGGGTATGCACCAGCTCCTGCACC |
| pRetrox-TRE3G-POC5-HA | For: ATCGATACGCGTGCGCCACCATGTCATCAGATGAGGAG<br>Rev: CCTGCACCTGCACCAGCTCCGTCAACCACTTTTATGGAATG |
| pRetrox-TRE3G-POC5$^{1-470}$-HA | For: ATCGATACGCGTGCGCCACCATGTCATCAGATGAGGAG<br>Rev: CCTGCACCTGCACCAGCTCCCACATACATTTCTTCTGATGC |
| pRetrox-TRE3G-POC5$^{266-575}$-HA | For: GATACGCGTGCGCCACCATGGTTTATGAAGGTAAACTAGCTG<br>Rev:CCTGCACCTGCACCAGCTCCGTCAACCACTTTTATGGAATG |
| pRetroX-TRE3G-POC5$^{\Delta 472-532}$-HA | For: GATACGCGTGCGCCACCATGCCAAGAGTTGTAACCTCTG<br>Rev: CCTGCACCTGCACCAGCTCCGTCAACCACTTTTATGGAATG<br><br>GGAGCTGGTGCAGGTGCA<br>CATGGTGGCGCACGCGTA |
| pRetroX-TRE3G-POC5$^{\Delta 152-184}$-HA | For: ATCGATACGCGTGCGCCACCATGTCATCAGATGAGGAG<br>Rev: CCTGCACCTGCACCAGCTCCGTCAACCACTTTTATGGAATG |
| pRetrox-TRE3G-FAM161A-HA | For: ATCGATACGCGTGCGCCACCatggccacctcccaccga<br>Rev: CCTGCACCTGCACCAGCTCCgtgtgattcttcaacagatttctcttcttcactttc |
| pRetrox-TRE3G-MDM1-HA | For: ATCGATACGCGTGCGCCACCATGCCGGTGCGCTTCAAG<br>Rev: CCTGCACCTGCACCAGCTCCTGTTTTACCCCAGAAATTCTCCTTC |
| pQPXIP-PGK-EGFP-POC1A | EGFP_Fragment: AGTCTAGCGGCCGCGCCACCATGGTGAGCAAGGGCGAG<br>CTTGTACAGCTCGTCCATGC<br><br>POC1A_FL_Fragment:<br>for: CATGGACGAGCTGTACAAGgcggccgcaGGAGCTGGTGCAGGTGCAGGAGCTGGTGCAGCTGCGCCCTGCGCGGAG<br>rev: TGGTGTTGCTCTCTGCATGATTAGCTGCTGG<br><br>Backbone: TCATGCAGAGAGCAACACCAtaaCTCGAGGAATTCCGCCCCCC<br>GGTGGCGCGGCCGCTAGA |

| | |
|---|---|
| pQPXIP-PGK-EGFP-POC1B | POC1B_Fragment:<br>For: CATGGACGAGCTGTACAAGgcggccgcaGGAGCTGGTGCAGGTGCAGGAGCTGGTGCAGCCTCAGCCACGGA<br>Rev: GCTTTTCTGTTGGACAGCACTGAA<br><br>Backbone_for: TTCAGTGCTGTCCAACAGAAAAGCtaaCTCGAGGAATTCCGCCCCCC<br>Backone_rev: GGTGGCGCGGCCGCTAGA |
| pRetrox_backbone | For: GGAGCTGGTGCAGGTGCAG<br>Rev: GGTGGCGCACGCGTATCG |
| pRetroX-TRE3G-POC1A-mNeonGreen | For: TCATGCAGAGAGCAACACCAGGAGCTGGTGCAGGTGCAGGAGCTGGTGCAATGGTGAGCAAGGGCGAG<br>Rev: TACCCGGTAGAATTCGGGCCttaCTTGTACAGCTCGTCCATGC<br><br>For: GGCCCGAATTCTACCGGG<br>Rev: TGGTGTTGCTCTCTGCATG |
| pRetroX-TRE3G-POC1B-mScarlet-I | For: GTGCTGTCCAACAGAAAAGCGGAGCTGGTGCAGGTGCA<br>Rev: TACCCGGTAGAATTCGGGCCTTACTTGTACAGCTCGTCCATGC<br><br>For:: GGCCCGAATTCTACCGGG<br>Rev:GCTTTTCTGTTGGACAGCAC |
| GBPsequence | For: gccgatgtgcagctggtg<br>Rev: tcatgaggagacggtgacc |
| POC1AKO_screen | KO_for: CACCATTCTCCTGCGTCAGC<br>KO_rev: TCCCAGCCTCTGCAAGACACCT<br>WT_for: TGCTCTGCTCCATGGGAGAC<br>WT_rev: cCAGGGTTCCTCCAAGGGTC |
| POC1BKO_screen | KO_for: TTGCTTTTTAATGGGGCGTGTG<br>KO_rev: CACACACCAGGGCCTGTTG<br>WT_for: TGTGTGCTAGAATGCAGAAATGCAGT<br>WT_rev: GTGGCAGGGGGAGGGATAGCAT |
| POC5KO_screen | KO_for: GAATGTCAGTAGCTAATTTGCAT<br>KO_rev: TTCCATAGCTCACATGAATATAC<br>WT_for: ATGATAGTTTCACCTCAGGCTG<br>WT_rev: TTAGCCAGGATGGTCTCGATCT |
| MultiBac | For: tctagagcctgcagtctcg<br>Rev: atatttataggtttttttattacaaaactg |
| POC5-HA_MB_for | taataaaaaaacctataaatatGTCATCAGATGAGGAGAAATAC |
| POC5-PL | For: GGAGCTGGTGCAGGTGCAG<br>Rev: GGTGGCGCACGCGTATCG |
| POC5-insert | For: ATCGATACGCGTGCGCCACCATGTCATCAGATGAGGAG<br>Rev: CCTGCACCTGCACCAGCTCCGTCAACCACTTTTATGGAATG |

**References**


1. Ko, J. M. *et al.* SOFT syndrome caused by compound heterozygous mutations of POC1A and its skeletal manifestation. *J Hum Genet* **61**, 561–564 (2016).
2. Roosing, S. *et al.* Disruption of the Basal Body Protein POC1B Results in Autosomal-Recessive Cone-Rod Dystrophy. *Am J Hum Genet* **95**, 131 (2014).
3. LI, S., ZHONG, Y., YANG, Y., HE, S. & HE, W. Further phenotypic features and two novel POC1A variants in a patient with SOFT syndrome: A case report. *Mol Med Rep* **24**, (2021).
4. Koparir, A. *et al.* Novel POC1A mutation in primordial dwarfism reveals new insights for centriole biogenesis. *Hum Mol Genet* **24**, 5378–5387 (2015).
5. Shaheen, R. *et al.* POC1A Truncation Mutation Causes a Ciliopathy in Humans Characterized by Primordial Dwarfism. *Am J Hum Genet* **91**, 330 (2012).
6. Al-Kindi, A. *et al.* A novel POC1A variant in an alternatively spliced exon causes classic SOFT syndrome: clinical presentation of seven patients. *J Hum Genet* **65**, 193–197 (2020).
7. Barraza-García, J. *et al.* Two novel POC1A mutations in the primordial dwarfism, SOFT syndrome: Clinical homogeneity but also unreported malformations. *Am J Med Genet A* **170**, 210–216 (2016).
8. Saida, K. *et al.* SOFT syndrome in a patient from Chile. *Am J Med Genet A* **179**, 338–340 (2019).
9. Kameya, S. *et al.* Phenotypical Characteristics of POC1B-Associated Retinopathy in Japanese Cohort: Cone Dystrophy With Normal Funduscopic Appearance. *Invest Ophthalmol Vis Sci* **60**, 3432–3446 (2019).
10. Yanık, Ö., Batıoğlu, F., Sahin, Y., Demirel, S. & Özmert, E. Seroreactivity against retinal proteins in a case of POC1B gene associated cone dystrophy with normal funduscopic appearance: a systematic approach to diagnosis. *Ophthalmic Genet* **44**, 389–395 (2023).
11. Jin, X. *et al.* Novel compound heterozygous mutation in the POC1B gene underlie peripheral cone dystrophy in a Chinese family. *Ophthalmic Genet* **39**, 300–306 (2018).
12. Gu, C., Li, J., Zhu, L., Lu, Z. & Huang, H. Analysis of catechol-O-methyltransferase gene mutation and identification of new pathogenic gene for paroxysmal kinesigenic dyskinesia. *Neurol Sci* **37**, 377–383 (2016).
13. Lawo, S. *et al.* HAUS, the 8-Subunit Human Augmin Complex, Regulates Centrosome and Spindle Integrity. *Current Biology* **19**, 816–826 (2009).
14. Elfmann, C. & Stülke, J. PAE viewer: a webserver for the interactive visualization of the predicted aligned error for multimer structure predictions and crosslinks. *Nucleic Acids Res* **51**, W404–W410 (2023).
15. Abramson, J. *et al.* Accurate structure prediction of biomolecular interactions with AlphaFold 3. *Nature 2024 630:8016* **630**, 493–500 (2024).
