# Supplementary material for: An interaction network of inner centriole proteins organised by POC1A-POC1B heterodimer crosslinks ensures centriolar integrity
Source: Nat Commun. 2024 Nov 14;15:9857. doi: 10.1038/s41467-024-54247-5 (PMC11564547; doi:10.1038/s41467-024-54247-5)
Supplement: Supplementary file 2 — Reporting Summary [file 41467_2024_54247_MOESM2_ESM.pdf]

nature portfolio

Corresponding author(s): Elmar Schiebel

Last updated by author(s): Nov 3, 2024

Reporting Summary

Nature Portfolio wishes to improve the reproducibility of the work that we publish. This form provides structure for consistency and transparency in reporting. For further information on Nature Portfolio policies, see our Editorial Policies and the Editorial Policy Checklist.

Statistics

For all statistical analyses, confirm that the following items are present in the figure legend, table legend, main text, or Methods section.

n/a	Confirmed	
☐	☒	The exact sample size (*n*) for each experimental group/condition, given as a discrete number and unit of measurement
☐	☒	A statement on whether measurements were taken from distinct samples or whether the same sample was measured repeatedly
☐	☒	The statistical test(s) used AND whether they are one- or two-sided<br>*Only common tests should be described solely by name; describe more complex techniques in the Methods section.*
☒	☐	A description of all covariates tested
☒	☐	A description of any assumptions or corrections, such as tests of normality and adjustment for multiple comparisons
☐	☒	A full description of the statistical parameters including central tendency (e.g. means) or other basic estimates (e.g. regression coefficient) AND variation (e.g. standard deviation) or associated estimates of uncertainty (e.g. confidence intervals)
☐	☒	For null hypothesis testing, the test statistic (e.g. *F*, *t*, *r*) with confidence intervals, effect sizes, degrees of freedom and *P* value noted<br>*Give P values as exact values whenever suitable.*
☒	☐	For Bayesian analysis, information on the choice of priors and Markov chain Monte Carlo settings
☒	☐	For hierarchical and complex designs, identification of the appropriate level for tests and full reporting of outcomes
☒	☐	Estimates of effect sizes (e.g. Cohen's *d*, Pearson's *r*), indicating how they were calculated

Our web collection on statistics for biologists contains articles on many of the points above.

Software and code

Policy information about availability of computer code

Data collection	Immunofluorescence data was acquired with softWoRx softWoRx v6.1.1 Release 5 (Applied Precision, GE) and Leica Falcon LAS X Flim v.3.5.7 (Leica Application Suite X). U-ExM images were processed with Huygens' Deconvolution software v18.10.0p7 (SVI Inc.). Immunoblots were acquired with LAS4000IR v2.1 software (FUJIFILM). Size exclusion chromatography (equipped either with Superdex 6 increase or with Superdex 75 column) was performed at Äkta go operated by Unicorn (version 7.5/7.9). Datasets for negative stain were collected at Talos L120C equipped with Ceta 16M using Thermofischer Scientific software EPU 2.9. Mass Photometry Data was recorded with Refeyn DiscoverMP 2024 R1 software. AlphaFold-Multimer (AF2, release version 2.3.2) with AF2 multimer_v3 parameter set and and the UniRef30 database version 2023_02 was used to perform predictions. Chain-interactions were determined with FoldX v5.0. The prediction of the POC5 tetramer in Paramecium was perfomed using AlphaFold3.
Data analysis	Immunofluorescence images and Immunoblots and coomassie blue-stained gels were analysed with Fiji (ImageJ Version 2.0.0/1.52p), FLIM-FRET data was analysed with and Leica Falcon LAS X Flim v.3.5.7 (Leica Application Suite X). Microsoft Excel (v16.90) and Prism (Graphpad version 10.2.3) were used for normalisation and significance tests and calculation of p-values and data representation. Protein structures were visualised using ChimeraX-1.6.1. Sequencing data of the knock out cell lines were analyzed with SnapGene v5.3.2. Negative stain EM data were processed using Relion 3.1, gCtf 1.06. Mass photometry data were analysed using Refeyn DiscoverMP 2024 R1 software (Refeyn Ltd, Oxford, UK).

For manuscripts utilizing custom algorithms or software that are central to the research but not yet described in published literature, software must be made available to editors and reviewers. We strongly encourage code deposition in a community repository (e.g. GitHub). See the Nature Portfolio guidelines for submitting code & software for further information.

Data

Policy information about availability of data

All manuscripts must include a data availability statement. This statement should provide the following information, where applicable:

- Accession codes, unique identifiers, or web links for publicly available datasets
- A description of any restrictions on data availability
- For clinical datasets or third party data, please ensure that the statement adheres to our policy

All the data that support the findings of this study are available in the Source Data file.

Research involving human participants, their data, or biological material

Policy information about studies with human participants or human data. See also policy information about sex, gender (identity/presentation), and sexual orientation and race, ethnicity and racism.

Reporting on sex and gender	No experiments with human participants were performed.
Reporting on race, ethnicity, or other socially relevant groupings	No experiments with human participants were performed.
Population characteristics	No experiments with human participants were performed.
Recruitment	No experiments with human participants were performed.
Ethics oversight	No experiments with human participants were performed.

Note that full information on the approval of the study protocol must also be provided in the manuscript.

Field-specific reporting

Please select the one below that is the best fit for your research. If you are not sure, read the appropriate sections before making your selection.

[x] Life sciences [ ] Behavioural & social sciences [ ] Ecological, evolutionary & environmental sciences

For a reference copy of the document with all sections, see nature.com/documents/nr-reporting-summary-flat.pdf

Life sciences study design

All studies must disclose on these points even when the disclosure is negative.

Sample size	For conventional luorescence microscopy experiments no statistical method was used to predict the sample size. For each technical replicate of an independent experiment at least 10 images were taken, each containing at least 5 cells to achieve sufficient number of cells and to avoid bias. For Expansion Microscopy of Knockout Cells for quantification of the protein distribution at least 10 Centrioles with near normal length were acquired. For FLIM-FRET experiments in living cells at least 6 cells per condition and independent experiment were used to avoid variation between cells. For negative stain EM data sample size was adapted to achieve enough particles for sufficient 2D class averaging. For wild type POC5/Centrin data a total of 521 micrographs were acquired and 109,299 particles were used for automatic picking. For the mutant POC5/Centrin 351 micrographs were acquired and 68,238 particles were used for picking.
Data exclusions	No data exclusions were performed. All collected negative stain EM images were used for particle picking and then Negative stain EM particle selection was performed in several 2D classification rounds and criterion was based on the shape of the class averages. For fluorescence microscopy experiments all images containing cells were used.
Replication	IF Experiments were repeated three times or at least two times where indicated in the figure legend. FLAG-IP and HA-IP experiments were repeated in three independent biologically experiments (for ratio quantification) and other immunoblots were repeated at least two times (N= 2 independent biologically experiment)s. Expansion Microscopy experiments were at least two times independently repeated. FLIM-FRET experiments were repated two times (N=2 biologically independent experiments). All immunofluorescence and immunoblot experiments were successful replicated. Protein purification, expression and SDS-analysis were perfomed at least two times. Negative stain EM data acquisition and Mass Photometry were performed once for each dataset with 0 repetitions. For Mass Photometry two measurements were taken (from diluted and undiluted sample).
Randomization	Positions for image acquisition in flourescence microscopy experiments were selected on the presence of cells and a wide field of view was used to image sufficent cell number to avoid biased image selection. Positions for image acquisition Negative stain EM were selected based on the presence of the particles. For ExM quantification of the distribution of porteins in the KO cell lines specifically centrioles with near-normal length were selected for comaprison with control cells. Other experiments were not related to randomization.
Blinding	For immunofluorescence experiments it was not feasible to apply blinding experiments because of large sample sizeas well as for Electron Microscopy experiments. In other cases, the findings were confirmed by independent approaches e.g. FLIM-FRET and IP experiments. Negative stain EM analysis and AlphaFold predictions were not blinded because it is performed computationally. For other experiments like cloning, expression protein purification it was technically not possible.

Reporting for specific materials, systems and methods

We require information from authors about some types of materials, experimental systems and methods used in many studies. Here, indicate whether each material, system or method listed is relevant to your study. If you are not sure if a list item applies to your research, read the appropriate section before selecting a response.

Materials & experimental systems

n/a	Involved in the study
☐	☒ Antibodies
☐	☒ Eukaryotic cell lines
☒	☐ Palaeontology and archaeology
☒	☐ Animals and other organisms
☒	☐ Clinical data
☒	☐ Dual use research of concern
☒	☐ Plants

Methods

n/a	Involved in the study
☒	☐ ChIP-seq
☒	☐ Flow cytometry
☒	☐ MRI-based neuroimaging

Antibodies

Antibodies used

Primary antibodies used in this study for IF and expansion microscopy were: γ-tubulin (mouse, 1:1000, abcam Ab27074), γ-tubulin (guinea pig, 1:50, homemade), PCNT (rabbit, 1:2000, abcam Ab4448), CEP97 (rabbit, 1:300, Bethyl A301-945A), PCNT (guinea pig, 1:800, homemade), CEP215 (rabbit, 1:500, Merck 06-1398), Centrin (mouse, 1:1000, Millipore MABC544), Centrin (rabbit, 1:500, Abcam ab101332), α-tubulin (mouse, 1:500, SigmaAldrich DM1A), α-tubulin (rabbit, 1:500, Proteintech 11224-1-AP), α-tubulin (mouse, 1:500, Proteintech 660311-1-Ig), HA tag (rat, 1:1000, Merck 11867423001), GFP (mouse, 1:1000, Roche 11814460001), POC1A (guinea pig, 1:200, homemade, Atorino et al. 2020), POC1A (rabbit, 1:300, PA5-59217, ThermoFisher), POC1B (guinea pig, 1:500, homemade, Atorino et al.2020), POC1B (rabbit, 1:250, PA5-24495, ThermoFisher), POC5 (rabbit, 1:1000, Bethyl A303-341A-T), FAM161A, (rabbit, 1:500, Sigma HPA-032119), WDR90 (rabbit 1:250, NovusBio, NBP2-31888), MDM1 (rabbit, 1:500, PA5-59638, ThermoFisher), CCDC15 (rabbit, 1:1000, ThermoFischer, PA5-59184), HAUS4 (rabbit, 1:500, Proteintech 20104-1-AP), CEP295 (rabbit, 1:500, Abcam Ab122490), CEP135 (rabbit, 1:200, homemade), CEP44 (rabbit, 1:500, homemade, Atorino et al. 2020). Mitosin (mouse, 1:100, BD biosciences)
Primary antibodies used in this study for IB were: FLAG tag (rabbit, 1:1000, Proteintech 20543-1-AP), HA tag (rat, 1:1000, Merck 11867423001), HA tag (rabbit, 1:1000, Proteintech 51064-2-AP), GAPDH (mouse, 1:1000, Proteintech 60004-1-lg), Vinculin (mouse, 1:5000, Proteintech 66305-1-lg), POC1A (guinea pig, 1:200, homemade, 17), POC1A (rabbit, 1:300, PA5-59217, ThermoFisher), POC1B (guinea pig, 1:500, homemade,17), POC1B (rabbit, 1:250, PA5-24495, ThermoFisher), POC5 (rabbit, 1:1000, Bethyl A303-341A-T).
Secondary antibodies used in this study for IF and expansion microscopy were: Anti-mouse IgG Alexa Fluor 488/555/647 (donkey, 1:500, ThermoFisher), Anti-rabbit IgG Alexa Fluor 488/555/647 (donkey, 1:500, ThermoFisher), Anti-guinea pig IgG Alexa Fluor 488/555/647 (goat, 1:500, ThermoFisher), Anti-rat IgG AlexaFluor 488/647 (donkey, 1:500, ThermoFisher), Anti-rabbit IgG Abberior STAR635P (goat, 1:500, Abberior) and Anti-mouse IgG Abberior STAR635P (goat, 1:500, Abberior).

Validation

Homemade CEP44, POC1A and POC1B antibodies were validated as previously described (Atorino et al. 2020). Commercial POC1A and POC1 antibody were validated with siRNA-treated cells and in the knockout cell lines. POC5 antibody was tested in siRNA treated cells and in the knockout cell line.
Anti-γ-tubulin (mouse, 1:1000, abcam Ab27074 LOT#GR317345-16):
https://www.abcam.com/gamma-tubulin-antibody-tu-30-ab27074.html
Anti-PCNT (rabbit, 1:2000, abcam Ab4448 LOT#GR3200989-1):
https://www.abcam.com/pericentrin-antibody-centrosome-marker-ab4448.html
Anti-CEP97 (rabbit, 1:300, Bethyl A301-945A LOT#n.s.):
https://www.bethyl.com/product/A301-945A/CEP97+Antibody
Anti-Centrin1 (mouse 1:1000, Millipore MABC544 LOT#2872235):
http://www.merckmillipore.com/DE/de/product/Anti-Centrin-Antibody-clone-20H5,MM_NF-04-1624?ReferrerURL=https%3A%2F%2Fwww.google.com%2F&bd=1
Anti-α-tubulin (mouse, 1:500, SigmaAldrich DM1A LOT#047M4789V):
https://www.sigmaaldrich.com/catalog/product/sigma/t9026?lang=de&region=DE
Anti-CEP295 (rabbit, 1:500, Abcam Ab122490 LOT#GR259105-2):
https://www.abcam.com/cep295-antibody-ab122490.html
Anti-GAPDH:
https://en.cellsignal.de/products/primary-antibodies/gapdh-14c10-rabbit-mab/2118
Anti-HA tag (rat, 1:1000, Sigma Aldrich 11867431001):
https://www.sigmaaldrich.com/catalog/product/roche/roahaha?lang=de&region=DE&gclid=EAIaIQobChMI0KTxtoCL5wIVS9HeCh32jwDnEAAYASAAEgJI1fD_BwE
CEP135 (rabbit, 1:100, previously described in doi: 10.1242/jcs.179713)
anti-Vinculin (https://www.ptglab.com/products/Vinculin-Antibody-66305-1-Ig.htm),
anti-DYKDDDDK (FLAG-tag) (https://en.cellsignal.de/products/primary-antibodies/dykddddk-tag-9a3-mouse-mab-binds-to-same-epitope-as-sigma-s-anti-flag-m2-antibody/8146),
anti-DDDDK tag (FLAG)(https://www.ptglab.com/products/Flag-Tag-Antibody-20543-1-AP.htm),

anti-α-tubulin
(https://www.sigmaaldrich.com/catalog/product/sigma/t9026?lang=de&region=DE) ,
Anti-CCDC15:
https://www.thermofisher.com/antibody/product/CCDC15-Antibody-Polyclonal/PA5-59184
Anti-FAM161A:
https://www.sigmaaldrich.com/DE/de/product/sigma/hpa032119
Anti-MDM1:
https://www.thermofisher.com/antibody/product/MDM1-Antibody-Polyclonal/PA5-59638
Anti-WDR90:
https://www.novusbio.com/products/wdr90-antibody_nbp2-31888
anti-α-tubulin:
https://www.ptglab.com/de/products/tubulin-Alpha-Antibody-66031-1-Ig.htm
anti-α-tubulin:
https://www.ptglab.com/de/products/TUBA1B-Antibody-11224-1-AP.htm
Anti-GFP:
https://www.sigmaaldrich.com/DE/de/product/roche/11814460001
Anti-CEP215:
https://www.merckmillipore.com/DE/de/product/Anti-CDK5RAP2-Antibody,MM_NF-06-1398
Anti-HAUS4:
https://www.ptglab.com/de/products/HAUS4-Antibody-20104-1-AP.htm
Anti-POC5:
https://www.biomol.com/de/produkte/antikoerper/primaerantikoerper/allgemein/anti-poc5-a303-341a-thaus4

Eukaryotic cell lines

Policy information about cell lines and Sex and Gender in Research

Cell line source(s)	RPE1 hTERT TRE3GV and HEK T293 as well as RPE1 CEP44KO cells were described in Atorino, E.S. et al. Nat Commun 11, 903 (2020). SF21 insect cell line obtained was obtained from EMBL protein expression facility.
Authentication	Cell lines were examined for their morphology by microscopy. Knockout cell lines were tested by immunoblot and immunofluorescence and were sequenced. Overexpression cell lines were tested by immunofluorescence using antibodies against the tag that was fused to the protein of interest.
Mycoplasma contamination	All cell lines (RPE1 hTERT TRE3GV and HEK T293 TRE3GV) and their derived cell strains were regularly tested for Mycoplasma and were found negative.
Commonly misidentified lines (See ICLAC register)	No commonly used misidentified cell lines were used.

Plants

Seed stocks	No experiments with plants were performed.
Novel plant genotypes	No experiments with plants were performed.
Authentication	No experiments with plants were performed.
